# Properties of Measurements of Patient‐Reported Outcome Measures Physical Activity Assessment in Chronic Kidney Disease: A Systematic Review

**DOI:** 10.1002/pri.70076

**Published:** 2025-06-10

**Authors:** Thamiê Cristina Stella, Inaê Silva Santos, Graziella Alves da Silva, Cid André Gomes, Luciana Maria Malosá Sampaio

**Affiliations:** ^1^ Postgraduate Program in Rehabilitation Sciences Universidade Nove de Julho São Paulo Brazil

**Keywords:** chronic kidney disease, physical activity, psychometric properties, systematic review

## Abstract

**Background and Purpose:**

The assessment of physical activity (PA) using Patient‐Reported Outcome Measures (PROMs) is routine due to its easy applicability and low cost. In the context of CKD, there are several studies that evaluate PA through PROM, but there is heterogeneity in the choice of tool. Therefore, the objective of this study was to identify, evaluate and synthesize the psychometric properties of PROMs used in the assessment of PA in CKD.

**Methods:**

A systematic review was carried out according to the PRISMA guidelines in PubMed, SciELO, Medline, Lilacs and EMBASE databases. The assessment of the quality of the studies was evaluated using the COSMIN Checklist and EMPRO tool.

**Results:**

15,137 studies were found, with 17 included. Eleven PROMs were found, of which 2 were elaborate to the CKD population: Chronic Kidney Disease Physical Activity Questionnaire (CKD‐PAQ) and Low Physical Activity Questionnaire (LoPAQ). Criterion validity was the most evaluated psychometric property. Only CKD‐PAQ shows satisfactory results in both evaluation tools for the psychometric properties evaluated.

**Discussion:**

This systematic review found no consensus in the literature for the best PROM for assessing PA in CKD. However, the CKD‐PAQ appears to be promising as the only PROM with a favorable evaluation by both COSMIN RoB and EMPRO. There is a lack of studies evaluating PA in the early stages of CKD and its responsiveness, indicating a gap in the research. COSMIN RoB and EMPRO differed from each other, showing that the choice of the evaluation tool must be guided by the evaluator's expertise and objective.

**Trial Registration:** Systematic review registered in PROSPERO (CRD42022312143)

## 1 Introduction

According to World Health Organization (WHO), physical activity (PA) is an important factor in the control and protection of chronic diseases (Bull et al. 2020). Chronic Kidney Disease (CKD) causes systemic alterations that impact the individual's functionality (Roshanravan et al. 2017), and the reduction in PA is proportional to renal function (Clarke et al. 2018). The disease is divided into six functional stages, where stage 1 is the mildest and stage 5 is the terminal phase requiring dialysis, and characterizing the most inactive part of CKD (KDIGO. Kidney Disease Improve Global Outcomes; Roshanravan et al. 2017). Stimulating PA in CKD brings benefits, such as increased survival, quality of life, and better control of comorbidities (Wilkinson et al. 2025).

Assessment of PA in CKD is particularly challenging. The gold standard method of measurement energy expenditure using doubly labeled water (DLW) is compromised in CKD due to impaired renal function in filtering isotopes (Sridharan et al. 2022). However, instruments objective PA assessment by wearable devices (e.g. accelerometers, pedometers and actigraphy) does not show good accuracy in distinguishing different levels of PA in predominantly inactive populations, such as in CKD (Sylvia et al. 2014).

The use of Patient‐Reported Outcome Measures (PROMs) for PA evaluation is routine due to its easy applicability and low cost (Sattler et al. 2021). In the CKD context, there are two specific PROMs published: the Low Physical Activity Questionnaire (LoPAQ) and the Chronic Kidney Disease Physical Activity Questionnaire (CKD‐PAQ). LoPAQ was created to discriminate PA levels in individuals on dialysis, the mostly sedentary group. However, it is limited by only covering the terminal phase of CKD (Johansen et al. 2015). In turn, CKD‐PAQ includes all stages of CKD (Sridharan et al. 2022), but it is a new PROM with no literature about applicability yet. For these reasons, generalist PROMs are still used in CKD, with heterogeneity in choice (Battaglia et al. 2024; Bakker et al. 2021).

Although the use of PROMs to assess PA in CKD is stimulating (Battaglia et al. 2024), the lack of uniformity in choice of PROM may compromise the practicability of the results. The COSMIN initiative (COnsensus‐based Standards for the selection of health Measurement Instruments) highlights the importance of systematic reviews of psychometric properties of PROMs for the standardization of clinical choice (Mokkink et al. 2016). Measurement properties (Validity, Reliability, and Responsiveness) are objective concepts that allow the evaluation of a subjective attribute, such as PA (Mokkink et al. 2016). Therefore, the objective of this study was to identify, evaluate and synthesize the psychometric properties of PROMs used in the assessment of PA in CKD.

## 2 Methods

This is a systematic review conducted according to PRISMA (Preferred Reporting Items for Systematic Reviews and Meta‐Analyses) (Page et al. 2021) guidelines and registered in PROSPERO (International Prospective Register of Systematic Reviews) under ID CRD42022312143. The PRISMA Checklist is available in Supporting Information S1: Table S1. Two independent reviewers conducted a parallel search and selection of studies in the PubMed, SciELO, Medline, Lilacs, and EMBASE databases.

As this study is a systematic review of measurement properties, the COSMIN initiative was used both to outline the research question and to choose descriptors (Prinsen et al. 2018). There were used four descriptors disease related (Chronic Kidney Disease; Chronic Renal Insufficiency; Chronic Kidney Failure; Dialysis), two construct related (Physical Activity; Physical Activities), and 12 descriptors psychometric related (Psychometrics; Patient Outcome Assessment; Outcome Assessment, Health Care; Reproducibility of Results; Reliability of Result; Validity of Result; Reliability and Validity; Test Retest Reliability; Validity, Face; Finding Reproducibility; Validation studies; Cross Cultural Comparison). The Boolean operators AND and NO were. The search strategy and the adaption for each database are available in Supporting Information S1: Tables S2 and S3, respectively.

The RAYYAN database was used for screening (Ouzzani et al. 2016). An indirect search was then carried out in the bibliographic references of the included studies, following the same screening. In case of disagreement among the evaluators, the resolution was carried out by consensus or by a third independent reviewer.

There were included studies with a sample of individuals aged 18 years or older, with CKD disease at any stage, whose clinical measure was PA and with the evaluation of at least one of the following properties of the instruments: validity, reliability and/or responsiveness. Abstracts of conference proceedings, literature reviews, and studies that did not use PROMs as the outcome variable for the analysis of physical assessment were excluded. No language restrictions were used.

The search was carried out between January and July 2022, with an update in May 2023 with the inclusion of the fourth descriptor disease related and in December 2024 due to evidence of new publications of interest.

### 2.1 Data Extraction

Data from the included articles were extracted independently by each reviewer. To standardize the process, a data extraction form was created based on PRISMA recommendations (Page et al. 2021) and extraction tables exemplified by COSMIN (Mokkink et al., 2017).

### 2.2 Psychometric Properties

The present study aimed to evaluate the psychometric variables commonly assessed in PROMs: reliability, validity and responsiveness.

Reliability is the degree or quality of consistency with which the instrument's items measure the construct and allows reproduction and obtaining consistent results when applied on different occasions (Echevarría‐Guanilo et al. 2017). It includes attributes of internal consistency (assessed by Cronbach's Alpha Coefficient—Coef*α*), measurement error (assessed by Standard Error of Measurement—SEM), test, and inter‐rater reliability (both evaluated by Intraclass Correlation Coefficient—ICC—or by Pearson or Spearman Correlation) (Luján‐Tangarife and Cardona‐Arias 2015).

Validity is the instrument's ability to measure the proposed construct for which it was designed (Echevarría‐Guanilo et al. 2017). It is composed of attributes such as face validity (no statistical evaluation, qualitative assessment), content validity (assessed by exploratory factor analysis—EFA), construct validity (assessed by Content Validity Index—CVI—and Content Validity Ratio—CVR), criterion validity and divergent/convergent validity (both evaluated by Pearson or Spearman Correlation) (Luján‐Tangarife and Cardona‐Arias 2015).

Responsiveness is the ability of the instrument to detect changes over time in the construct being measured, and it's often utilized to evaluate disease progression or treatment response (Echevarría‐Guanilo et al. 2017).

### 2.3 Assessment of the Quality of Studies

To assess the quality of the studies, we used the COSMIN Risk of Bias (COSMIN RoB) Checklist (Mokkink et al., 2017) and the EMPRO (Evaluating Measures of Patient‐Reported Outcomes) tool (Valderas et al. 2008). The application of the instruments was carried out independently by each reviewer, and cases of disagreement were resolved by means of consensus or evaluation by a third reviewer.

The choice of tools was based on a previous study that verified the performance of different psychometric assessment tools (Lorente et al. 2020): while COSMIN is the most widespread tool to assess measurement properties of PROMs health related; EMPRO tool, on the other hand, is less complex and includes a more thorough assessment of attributes such as feasibility, application burden and interpretability, attributes that are not explored in COSMIN (Lorente al. 2020).

#### 2.3.1 Checklist COSMIN RoB

The COSMIN RoB checklist has 10 sections, with 4–35 items each, which evaluate: Reliability (contains the measurement properties internal consistency, reliability and error measurement), Validity (contains the content validity, criterion validity, and construct validity properties), and Responsiveness (Mokkink et al. 2017). Each measure property aspect evaluates to “+” (sufficient), “−” (insufficient), or “?” (undetermined). Then, the measurement property is classified according to the risk of bias, inconsistency, imprecision, and ambiguity. In the end, the quality of the property is rated as “High”, “Moderate”, “Low” or “Very Low” (Mokkink et al. 2020).

The complete tool and manuals are available for free access and use in the English language (Mokkink et al. 2020).

#### 2.3.2 EMPRO Tool

The EMPRO tool was developed by Valderas et al. (2008) and has 39 items distributed in 8 attributes: Conceptual and measurement model; Reliability; Validity; Responsiveness; Interpretability; Overload; Alternative management models, Linguistic and cross‐cultural adaptations. For each item, there is a 4‐point Likert scale of agreement, where “4” is “Strongly agree” and 1 is “Strongly disagree”. The score can reach from “0” (worst score) to “100” (best score), with values equal or greater than “50” considered acceptable for the attribute (Valderas et al. 2008).

The EMPRO tool requires a license application through the electronic portal https://bibliopro.org and is available in English and Spanish. There are still few published studies using it and none evaluating PROMs of PA.

## 3 Results

**FIGURE 1:**
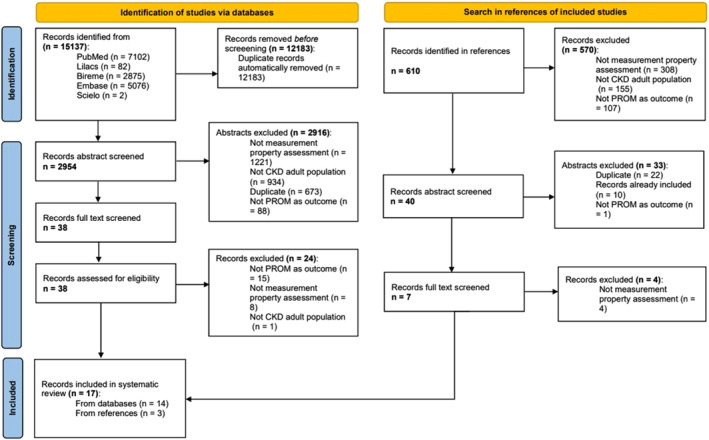
PRISMA flow chart.

In total, 15,137 articles were found, of which 14 were selected for inclusion in the systematic review. In the secondary search of the bibliographic references of the included articles, there was the inclusion of another 3 articles not found in the initial search, totaling 17 articles selected for evaluation (Figure 1). The year of publication ranged from 2001 to 2024, with a peak of publication in 2019 (3 studies). All studies were published in English. The Table 1 shows the description of the included studies.

**TABLE 1:** Methodological characteristics of the included studies.

Study	Study design	Population	*n*	PROM(s)	Psychometric properties	Comparative test	Statistics	Cutoff points	Outcomes
Hadjiioannou et al. (2019)	Controlled, non‐randomized, single‐center pragmatic clinical trial	Non‐dialysis and dialysis CKD Renal transplant (excluded from this evaluation)	40 individuals (total), but 12 transplanted. Only 28 individuals evaluated in this review Age (mean): 56.7 years Gender: 20 males Sample wasn't calculated	DASI	*Reliability* – Absolute – Test‐retest	NA	*Absolute reliability:* SEM e MCID *Test‐retest reliability:* ICC	ND	*Non‐dialysis CKD:* SEM = 3.95 MCID = 9.21 ICC = 0.9 *Dialysis CKD:* SEM = 3.0 MCID = 8.57 ICC = 0.87
Hatef et al. (2018)	Cross‐sectional observational study	CKD dialysis (HD)	404 individuals, among which 202 selected for exploratory statistics and 202 selected for confirmatory statistics Age (mean): 56.8 ± 12.04 years Gender: 58% male Sample was calculated	ESES (Persian language)	*Validity* – Face – Content – Construct (structural) – Convergent *Reliability* – Construct – Internal consistency *Cross‐cultural adaptation from* Persian language	NA	*Face validity:* Qualitative assessment *Content validity:* CVR and CVI *Construct validity:* EFA *Convergent validity:* AVE *Internal consistency:* Coefα, coefθ, CoefΩ *Construct reliability:* Coefα	*CVR* > 0.49 *CVI* > 0.79 *EFA*: – *x*2 > 0.05 – AGFI > 0.8 – *RMSEA* > 0.08 *AVE* > 0.5 *Coefficients* > 0.7	*CVR* and *CVI* not described *EFA* = – *x*2 = 106.70 – AGFI = 0.76 – RMSEA = 0.06 *AVE* = 0.631 *Coefα* = 0.94 *Coefθ* = 0.96 *CoefΩ* = 0.91 *Construct reliability* = 0.894
Huang et al. (2021)	Multicenter cross‐sectional observational study	CKD dialysis (HD)	85 individuals, among which 29 took the test twice for test‐retest evaluation Age (mean): 62.3 ± 11.8 years Gender: 48 males Sample wasn't calculated	*LoPAQ* (Mandarin language) Subdivided in six categories: Sitting, walking, light activity, moderate activity, vigorous activity and total activity	*Validity* – Content – Criterion *Reliability* – Internal consistency – Test‐retest *Cross‐cultural adaptation* from mandarin language	Actigraphy by *ActiGraph GT3X +* for 7 days	*Content validity:* CVR and CVI *Criterion validity:* Spearman's correlation *Internal consistency:* Coefα *Test‐retest reliability:* ICC	*CVI* > 0.78 *CVR* > 0.75 *Spearman* > 0.40 *ICC:* Excellent > 0.75 Good: 0.60—0.74 Fair: 0.40—0.59 Poor < 0.40	*CVI* **=** 0.91 *CVR* **=** 0.98 *Accelerometer correlations:* Sitting = 0.22** Walking = 0.47* Total activity = 0.44* *ICC:* Sitting = 0.43 Walking = 0.30 Light activity = 0.49 Moderate activity = 0.59 Vigorous activity = 0.66 Total activity = 0.47 *Coefα* not described
Johansen et al. (2001)	Cross‐sectional and multicenter observational study	CKD dialysis (HD)	39 individuals, among which 29 took the test twice for test‐retest evaluation Age (mean): 52 ± 16 years Gender: 26 males Sample wasn't calculated	*HAP* Subdivided in two scores: MAS (maximum activity score) and AAS (adjusted activity score) *PAR* *PASE*	Criterion validity Test‐retest reliability	Accelerometer *TriTrac‐R3D* for 7 days Physical performance through assessment of time for activities	*Criterion validity:* Pearson's correlation Test‐retest reliability: Not specified	ND	*HAP‐MAS:* Accelerometer correlation = 0.78* Performance assessment correlation = 0.72* *HAP‐AAS:* Accelerometer correlation = 0.,73* Performance assessment correlation = 0.75* Test‐retest reliability = 0.91 *PASE:* Accelerometer correlation = 0.66* Performance assessment correlation = 0.50* *PAR:* Accelerometer correlation = 0.59* Performance assessment correlation = 0.36**
Johansen et al. (2015)	Cross‐sectional and multicenter observational study	CKD dialysis (HD)	68 individuals Age (mean): 59.0 ± 14 years Gender: 59% male Sample wasn't calculated	*LoPAQ* Subdivided in walking and total activity	*Validity* – Construct (hypothesis testing) – Convergent	Short form (36) health survey (SF‐36), physical component Short physical performance battery (SPPB) Minnesota leisure time activities questionnaire (LTA)	Spearman's correlation	ND	*Correlations with LTA:* Walking = 0.58* Total activity = 0.62* *Correlation with SF‐36* **=** 0.64* *Correlation with SPPB* **=** .51*
Kittiskulnam et al. (2019)	Cross‐sectional and multicenter observational study	CKD dialysis (HD and PD)	60 individuals (48 on HD and 12 on PD) Age (mean): 58,0 ± 12,7 years Gender: 78,3% male Sample wasn't calculated	*LoPAQ* Subdivided in walking and total activity	*Validity* – Construct (hypothesis testing) – Criterion	Short form (36) health survey (SF‐36), physical component Short physical performance battery (SPPB) Pedometer *Accusplit AE 120* TriTrac‐R3D for 7 days	Pearson's correlation	ND	*Pedometer correlations:* Walking = 0.53* Total activity = 0.35* *Correlation with SF‐36* **=** 0.29** *Correlation with SPPB* **=** 0.26** No statistical difference between dialysis modalities
Lou & He, (2019)	Multicenter cross‐sectional observational study	CKD dialysis (HD)	320 individuals, among which 103 took the test twice for test‐retest evaluation. Age (mean): 58.6 years Gender: 200 males Sample wasn't calculated	*IPAQ* short version (Mandarin language) Subdivided in four categories: Walking, moderate activity, vigorous activity and total activity	Criterion validity Test‐retest reliability	Pedometer OMRON HJ‐328 utilized for 7 days	*Criterion validity:* Spearman's correlation *Test‐retest reliability:* ICC	*Spearman:* Large: 0.8 Moderate: 0.5 Small: 0.2 *ICC:* Not specified	*Pedometer correlations:* Walking = 0.407* Moderate activity = 0.43* Vigorous activity = 0.28* Total activity = 0.561* *ICC:* Walking = 0.90* Moderate activity = 0.62* Vigorous activity = 0.99* Total activity = 0.84*
Overend et al. (2010)	Observational test‐retest and single‐center study	CKD dialysis (HD)	25 individuals Age (mean): 67.2 years Gender: 14 males Sample was calculated	*HAP* (MAS and AAS)	*Reliability* – Absolute – Test‐retest	NA	*Absolute reliability:* SEM and MCID *Test‐retest reliability:* ICC	*SEM* calculated from standard deviation and ICC *MCID* calculated from SEM *ICC* **>** 0.8	*HAP‐MAS:* SEM = 5.5 MCID = 15.1 ICC = 0.76 *HAP‐AAS:* SEM = 4.1 MCID = 11.4 ICC = 0.92
Ravani et al. (2012)	Cross‐sectional, single‐center observational study	Non‐dialysis CKD (stages 3 and 4)	43 individuals, among wich 23 for reliability assessment and 20 for validity assessment. Ages (mean): 58.0 ± 12.7 years Gender: 78.3% male Sample was calculated	DASI	Test‐retest reliability Criterion validity	Cardiopulmonary exercise test (CPET)	*Test‐retest reliability:* ICC *Criterion validity:* CCC	ND	*ICC:* CKD stage 3 = 0.71 CKD stage 4 = 0.81 *CCC* **=** 0.67
Robinson‐Cohen et al. (2013)	Prospective single‐center cohort study	Non‐dialysis CKD	46 individuals Age (mean): 55 ± 11 years Gender: 54% male Sample wasn't calculated	*FWH* *HAP* (MAS and AAS) *IPAQ* (only 2 questions about sitting time) *PASE*	Criterion validity	Actigraphy by *ActiGraph GT3X +* for 14 days	Pearson's correlation	ND	*PASE* **=** 0.36* *FWH* **=** 0.28* *HAP‐MAS* **=** 0.56* *HAP‐AAS* **=** 0.49* *IPAQ* **=** −0.26**
Rosa et al. (2015)	Multicenter cross‐sectional observational study	CKD dialysis (HD)	40 individuals Age (mean): 54 ± 16 years Gender: 21 females Sample wasn't calculated	*IPAQ* short version (brazilian Portuguese language) Subdivided in light activity, moderate to vigorous activity and total activity	Criterion validity	Actigraphy by *ActiGraph GT3X +* for 14 days	Spearman's correlation	ND	Light activity = 0.34** Moderate to vigorous activity = 0.38** Total activity = 0.41**
Sridharan et al. (2016)	Prospective cross‐sectional study	Non‐dialysis CKD	40 individuals, among which 21 from stages 1–3 and 19 from stages 4–5. Age (mean): 54.1 ± 17.4 years Gender: 22 males Sample wasn't calculated	*PAR* *RPAQ*	Criterion validity	Total energy expenditure (TEE) from the double labeled water test.	Bland‐altman test	ND	*Correlation with TEE:* RPAQ = 0.57 PAR = 0.23
Sridharan et al. (2022)	Longitudinal cohort study	CKD all stages	*Development phase:* 266 individuals, among which 40 for qualitative assessment Age (mean): 58.3 ± 15.1 years Gender: 57.6% males *Final phase:* 523 individuals Age (mean): 60.8 ± 16.1 years Gender: 63.7% male Sample wasn't calculated	*CKD‐PAQ* – MET value – TEE value	*Validity* – Construct (hypothesis testing) in development phase – Convergent in final phase	Accelerometer *ActiGraph GT9X +* Link for 7 days RPAQ questionnaire	ICC and bland‐altman test	ND	*Development phase:* ICC (accelerometer): MET value = 0.45* TEE value = 0.75* ICC (RPAQ): MET value = 0.90* TEE value = 0.91* *Final phase:* ICC (RPAQ): MET value = 0.83* TEE value = 0.92*
Tabib et al. (2024)	Cross‐sectional study	CKD dialysis (HD)	109 individuals Age (mean): 64 ± 11 years Gender: 56% male Sample wasn't calculated	LoPAQ (Persian language)	*Validity* – Content – Convergent *Test‐retest* reliability *Cross‐cultural adaptation* from Persian language	Community healthy adults model program for seniors (CHAMPS) questionnaire Short form (36) health survey (SF‐36), physical component Short physical performance battery (SPPB)	*Content validity:* CVI *Convergent validity:* Spearman's correlation *Test‐retest reliability:* ICC	*CVI* > 0.78 *Spearman* > 0.40 *CCI:* Excellent > 0.75 Good 0.60–0.74 Fair 0.40–59 Poor < 0.40	*CVI* **=** 0.86 *Correlation with CHAMPS questionnaire* **=** 0.85* *Correlation with SF‐36* **=** 0.7** *Correlation with SPPB* **=** 0.67** *ICC* **=** 0.65–0.78
Wellard (2003)	Cross‐sectional observational study	CKD dialysis (HD)	65 individuals Age (mean): 62 ± 13.4 years Gender: 47 males Sample wasn't calculated	*HAP* (AAS)	Convergent validity	Sickness impact profile (SIP) questionnaire	Spearman's correlation	ND	*Correlation with SIP (physical component)* **=** 0.81* *Correlation with SIP (total)* **=** 0.76*
Wilkinson et al. 2022	Secondary analysis of the research group's database of previous studies	CKD all stages	40 individuals, among which 11individuals for test‐retest reliability assessment Age (mean): 62.5 ± 11.1 years Gender: 21 females Convenience sample	GPPAQ	Criterion validity Test‐retest reliability	Accelerometer GENEActiv for 7 days	*Criterion validity:* Sensitivity specificity Positive predictive value (PPV) and negative predictive value (NPV) *Test‐retest reliability:* Weighted kappa (kw)	ND	Sensitivity = 54.5% Specificity = 96.6% PPV = 85.7% NPV = 84.9% Accuracy = 85% Kw = 0.74
Yamabe et al. (2023)	Cross‐sectional observational study	CKD dialysis (HD)	220 individuals Age (mean): 67.8 ± 11.6 years Gender: 59.1% male Sample wasn't calculated	*LoPAQ* (Japanese language) Subdivided in sitting, walking and total activity	Criterion validity Cross‐cultural adaptation from Japanese language	Pedometer *Lifecorder*, SUZUKEN co. Ltd. For 10–14 days	*Criterion validity:* Spearman's correlation	*Spearman:* Large: 0.8 moderate: 0.5 Small: 0.2	*Pedometer correlations:* Sitting = −0.14** Walking = 0.53* Total activity = 0.49*

*Note:* Numeric data presented as Mean ± Standard Deviation or %.

Abbreviations: AGFI, adjusted goodness‐of‐fit index; AVE, average variance extracted; CCC, concordance correlation coefficient; CKD, chronic kidney disease; Coefα, Cronbach's *α* coefficient; Coefθ, *θ* coefficient; Coef*Ω*, McDonald's *Ω* coefficient; CVI, content validity index; CVR, content validity ratio; EFA, exploratory factor analysis; HD, hemodialysis; ICC, intraclass correlation coefficient; MCDI, minimum clinically important difference; MET, metabolic equivalent of task; NA, not applicable; ND, not described; PD, peritoneal dialysis; RMSEA, root mean square error of approximation; SEM, standard error of measurement; TEE, total energy expenditure; x2, chi‐square.

*p < 0.001.

**p < 0.05.

Eleven different PROMs were found, which only 2 are specific for CKD patients: CKD‐PAQ (Sridharan et al. 2022) and LoPAQ (Sridharan et al. 2022; Huang et al. 2021; Johansen et al. 2015; Kittiskulnam et al. 2019; Tabibi et al. 2024). In non‐specific PROMs, were found evaluation of Duke Activity Status Index (DASI) (Yamabe et al. 2023; Hadjiioannou et al. 2019), Exercise Self‐Efficacy Scale (ESES) (Ravani et al. 2012), Four Week Physical History Questionnaire (FWH) (Hatef et al. 2018), General Practice Physical Activity Questionnaire (GPPAQ) (Robinson‐Cohen et al. 2013), Human Activity Profile (HAP) (Wilkinson et al. 2022; Johansen et al. 2001; Overend et al. 2010; Robinson‐Cohen et al. 2013), International Physical Questionnaire (IPAQ) (Wellard 2003; Lou and He 2019; Robinson‐Cohen et al. 2013), Stanford 7‐Day Physical Activity Recall (PAR) (Rosa et al. 2015; Johansen et al. 2001), Physical Activity Scale for the Elderly (PASE) (Sridharan et al. 2016; Johansen et al. 2001) and Recent Physical Questionnaire (RPAQ) (Robinson‐Cohen et al. 2013).

All stages of CKD were included, but with emphasis on stages 3b–5. ESES and LoPAQ presented the evaluation of the highest number of measurement properties. The most studied psychometric property was criterion validity, in 10 (58%) studies (Sridharan et al. 2016; Huang et al. 2021; Johansen et al. 2001; Kittiskulnam et al. 2019; Lou and He 2019; Ravani et al. 2012; Robinson‐Cohen et al. 2013; Rosa et al. 2015; Sridharan et al. 2016; Wilkinson et al. 2022). The main instrument of comparison was actigraphy, in 3 studies (18%) (Yamabe et al. 2023; Huang et al. 2021; Robinson‐Cohen et al. 2013). Only 1 (6%) study used the DLW method (Rosa et al. 2015).

The evaluation of the methodological quality of the articles by COSMIN RoB and EMPRO is available in Supporting Information S1: Tables S4 and S5. The main reasons that reduced the quality of the studies were small sample size, unsatisfactory numerical values of the results, and incomplete methodological description. In summarization of the level of evidence according to the COSMIN RoB (Table 2), the best performances in validity were obtained in the CKD‐PAQ for construct and convergent validity and in HAP questionnaire for convergent validity. For reliability, DASI and IPAQ presented sufficient results, while ESES presented satisfactory results in the evaluation of internal consistency.

**TABLE 2:** Summary of the PROMs evidence level by COSMIN RoB.

PROM	Psychometric properties								
	Construct validity (structural)	Construct validity (hypotheses testing)	Content validity	Criterion validity	Convergent validity	Cross‐cultural validity	Internal consistency	Reliability	Measurement error
*CKD*‐*PAQ* Sridharan et al. (2022)	—	(+) Moderate	—	—	(+) Moderate	—	—	—	—
*DASI* Hadjiioannou et al. (2019); Ravani et al. (2012)	—	—	—	(−) Low	—	—	—	(+) Moderate	(+) Low
*ESES* Hatef et al. (2018)	(−) High	—	(?) Low	—	(−) High	(?)^a^ Moderate	(+) High	(+) Low	—
*FWH* Robinson‐Cohen et al. (2013)	—	—	—	(−) Low	—	—	—	—	—
*GPPAQ* Wilkinson et al. (2022)	—	—	—	(±) Low	—	—	—	(+) Low	—
*HAP* Johansen et al. (2001); Overend et al. (2010); Robinson‐Cohen et al. (2013); Wellard (2003)	—	—	—	(±) Moderate	(+) Moderate	—	—	(+) Low	(+) Low
*IPAQ* Lou & He (2019); Robinson‐Cohen et al. (2013); Rosa et al. (2015)	—	—	—	(−) High	—	—	—	(+) Moderate	—
*LoPAQ* Johansen et al. (2015); Huang et al. (2021); Kittiskulnam et al. (2019); Tabibi et al. (2024); Yamabe et al. (2023)	—	(−) High	(+) Low	(±) High	(−) High	(?)^a^ ^,^ ^b^ ^,^ ^c^ Very low	(?) Very low	(±) Moderate	—
*PAR* Johansen et al. (2001); Sridharan et al. (2016)	—	—	—	(−) Moderate	—	—	—	—	—
*PASE* Johansen et al. (2001); Robinson‐Cohen et al. (2013)	—	—	—	(−) Moderate	—	—	—	—	—
*RPAQ* Sridharan et al. (2016)	—	—	—	(−) Low	—	—	—	—	—

*Note:* (+), Sufficient; (−), Insufficient; (±), Inconsistent; (?), Indeterminate.

^a^ Regarding cross‐cultural validity in the Persian language.

^b^ Regarding cross‐cultural validity in the mandarin language.

^c^ Regarding cross‐cultural validity in the japanese language.

In summarization of the results using the EMPRO tool (Table 3), only CKD‐PAQ and GPPAQ presented satisfactory validity values. DASI, ESES, GPPAQ, IPAQ and HAP questionnaires showed acceptable reliability values. COSMIN RoB and EMPRO tool agreement in the evaluation of CKD‐PAQ, DASI, FWH, PAR, PASE and RPAQ. Among them, only CKD‐PAQ presented satisfactory evidence in the properties evaluated by the two tools (Table 4).

**TABLE 3:** Summary of the PROMs psychometric properties assessment by EMPRO tool.

PROM	EMPRO attributes									
	Conceptual and measurement	Reliability (internal consistency)	Reliability (reproducibility)	Validity	Responsiveness	Interpretability	Burden (respondent)	Burden (administrative)	Alternative modes of administration	Cultural and language adaptations
*CKD*‐*PAQ* Sridharan et al. (2022)	**52**.**4** *	—	—	**50**.**0** *	—	**55**.**5** *	—	—	—	—
*DASI* Hadjiioannou et al. (2019); Ravani et al. (2012)	—	33.3	**66**.**6** *	22.2	—	**55**.**5** *	—	—	—	—
*ESES* Hatef et al. (2018)	**71**.**4** *	**58**.**3** *	16.7	38.9	—	—	22.2	22.2	—	**100**.**0** * ^,^ ^a^
*FWH* Robinson‐Cohen et al. (2013)	—	—	—	22.2	—	44.4	—	—	—	—
*GPPAQ* Wilkinson et al. (2022)	—	NA	**58**.**3** *	**50**.**0** *	—	**66**.**6** *	—	—	—	—
*HAP* Johansen et al. (2001); Overend et al. (2010); Robinson‐Cohen et al. (2013); Wellard (2003)	—	16.6	**66**.**6** *	12.9	—	**51**.**8** *	—	—	—	—
*IPAQ* Lou & He (2019); Robinson‐Cohen et al. (2013); Rosa et al. (2015)	—	NA	**50**.**0** *	12.9	—	44.4	—	—	—	—
*LoPAQ* Johansen et al. (2015); Huang et al. (2021); Kittiskulnam et al. (2019); Tabibi et al. (2024); Yamabe et al. (2023)	**66**.**7** *	8.3	29.1	39.9	—	**61**.**0** *	—	—	—	**66**.**6** * ^,^ ^a^ ^,^ ^b^ ^,^ ^c^
*PAR* Johansen et al. (2001); Sridharan et al. (2016)	—	NA	33.3	13.9	—	**66**.**6** *	—	—	—	—
*PASE* Johansen et al. (2001); Robinson‐Cohen et al. (2013)	—	NA	33.3	13.9	—	49.9	—	—	—	—
*RPAQ* Sridharan et al. (2016)	—	—	—	22.2	—	**77**.**7** *	—	—	—	—

Abbreviation: NA: Not Applicable.

*Values above the acceptable limit.

^a^ Refers transcultural validity to the Persian language.

^b^ Refers transcultural validity to the Mandarin language.

^c^ Refers transcultural validity to the japanese language.

**TABLE 4:** Comparison of results obtained by COSMIN RoB and EMPRO tools.

PROM	Assessment tools	Psychometric properties					
		Desing and concept	Validity	Reliability	Responsiveness	Interpretability	Cross‐cultural validity
*CKD*‐*PAQ* Sridharan et al. (2022)	COSMIN RoB EMPRO	**✔** **✔**	**✔** **✔**	—	—	— **✔**	—
*DASI* Hadjiioannou et al. (2019); Ravani et al. (2012)	COSMIN RoB EMPRO	—	**✖** **✖**	**✔** **✔**	—	— **✔**	—
*ESES* Hatef et al. (2018)	COSMIN RoB EMPRO	**✖** **✔**	**✖** **✖**	**✔** **✔**	—	—	**✖** **✔**
*FWH* Robinson‐Cohen et al. (2013)	COSMIN RoB EMPRO	—	**✖** **✖**	—	—	— **✖**	—
*GPPAQ* Wilkinson et al. (2022)	COSMIN RoB EMPRO	—	**✖** **✔**	**✖** **✔**	—	— **✔**	—
*HAP* Johansen et al. (2001); Overend et al. (2010); Robinson‐Cohen et al. (2013); Wellard (2003)	COSMIN RoB EMPRO	—	**✔** **✖**	**✖** **✔**	—	— **✔**	—
*IPAQ* Lou & He (2019); Robinson‐Cohen et al. (2013); Rosa et al. (2015)	COSMIN RoB EMPRO	—	**✖** **✖**	**✔** **✖**	—	— **✖**	—
*LoPAQ* Johansen et al. (2015); Huang et al. (2021); Kittiskulnam et al. (2019); Tabibi et al. (2024); Yamabe et al. (2023)	COSMIN RoB EMPRO	**✖** **✔**	**✖** **✖**	**✖** **✖**	—	— **✔**	**✖** **✔**
*PAR* Johansen et al. (2001); Sridharan et al. (2016)	COSMIN RoB EMPRO	—	**✖** **✖**	—	—	— **✔**	—
*PASE* Johansen et al. (2001); Robinson‐Cohen et al. (2013)	COSMIN RoB EMPRO	—	**✖** **✖**	—	—	— **✖**	—
*RPAQ* Sridharan et al. (2016)	COSMIN RoB EMPRO	—	**✖** **✖**	—	—	— **✔**	—

*Note:* **✔** Satisfactory result: sufficient and high or moderate quality result summarization by COSMIN RoB, OR summary value equal or greater than 50.0 by EMPRO; **✖** Unsatisfactory result: insufficient or inconsistent result summary OR sufficient, but with low or very low quality by COSMIN RoB; OR summary value less than 50.0 by EMPRO.

## 4 Discussion

The present study is the first systematic review of psychometric assessment of PA PROMs in CKD. The literature already has systematic reviews in CKD in the psychometric evaluation of clinical tests of physical function and PROMs for the evaluation of quality of life and symptoms (Sridharan et al. 2016; Aiyegbusi et al. 2017), but not of PA itself. In this review, the evidence found is relatively recent (from 2001 to 2024), with few studies (17 in total) and with great heterogeneity in choice of PROM (11 different PROMs), psychometric assessment and methodological quality.

In CKD, although there is evidence of the importance of maintaining PA, the assessment and determination of clinically important levels still lacks documented clarity (MacRae et al. 2023). Wilkinson et al. (2025) pointed out that the functional assessment of individuals with CKD was not routine, probably due to the lack of guidelines on which tests to use. Thus, this study has clinical relevance for evaluating which PA PROMs are used in CKD and their performance in this population group.

Validity was evaluated in all PROMs found. This finding is expected, considering that validity investigates whether the instrument evaluated has the capacity to measure the intended construct (Bakker et al. 2021). On the other hand, reliability was evaluated in only 6 (54%) PROMs. Reliability investigates the instrument's ability to measure the construct without inaccuracies (Echevarría‐Guanilo et al. 2017). Therefore, the available evidence seeks to support the use of PA PROMs in the context of CKD but requires further research on precision.

The choice to assess the criterion validity is also understandable. This attribute compares the instrument of interest with a widely accepted and used clinical tool (Echevarría‐Guanilo et al. 2017). The comparison of PA PROMs with objectives and wearable dispositives brings clinical relevance to the findings of the studies included in this review. However, these dispositives are reporting low sensitivity in differentiating activity levels in predominantly inactive populations (Echevarría‐Guanilo et al. 2019). This explains the poor performance of most PROMs in assessing validity but creates a debate about which is the best comparison instrument for validating.

Among the non‐CKD‐specific PROMs, HAP had the best performance in the evaluation of validity and reliability. This is probably because the PROM was originally designed for Chronic Obstructive Pulmonary Disease (COPD) (Sylvia et al. 2014), a chronic disease that leads to functional impairment. We hypothesized that HAP had better performance because it was designed for a mostly inactive target population. The Italian Society of Nephrology cites HAP as an option to assess PA in CKD, but highlights that the PROM's length PROM (94 items) may be a limitation for clinical use (Fix and Daughton 1988).

Regarding CKD‐specific PROMs, all indexed literature about them was included in this review. LoPAQ was first described by Bakker et al. (2021) and has 5 published studies (Johansen et al. 2015; Huang et al. 2021; Johansen et al. 2015; Kittiskulnam et al. 2019; Tabibi et al. 2024) and CKD‐PAQ was first published by Yamabe et al. (2023). While CKD‐PAQ proposes to be a PROM more comprehensive and to assess, screen, and prevent the functional decline of milder staging of the disease, LoPAQ, seeks to better distinguish PA levels between dialysis patients, who suffer greater functional impact.

In this review, LoPAQ showed unsatisfactory performance in all properties evaluated. The authors of the studies point out the similarity of these results with other studies that evaluated and validated PROMs against objective dispositives (Sridharan et al. 2022; Huang et al. 2021). Therefore, the insufficient results of LoPAQ found in these studies are not capable of excluding the clinical relevance of this PROM but show the need to validate the LoPAQ with instruments more sensitive to sedentary profile. It is noteworthy that LoPAQ also presented inconsistent results for reliability. Only two studies investigated the measurement property (Kittiskulnam et al. 2019; Huang et al. 2021), with differences in sample number and interval time for test‐retest. Hence, it is necessary that the reliability of LoPAQ be studied carefully.

In summary of the results, only the CKD‐PAQ presented satisfactory results in both COSMIN RoB and EMPRO tools. However, some points need to be taken into consideration. First, there is only one study available and only validity property was evaluated (Tabibi et al. 2024), still requiring reliability assessment. Furthermore, validity was assessed based on the PROM from which the CKD‐PAQ was developed (RPAQ), making it necessary to evaluate the CKD‐PAQ with other comparative instruments.

Most of the studies have focused on severe stages of CKD. Although functional decline is more noticeable in the most severe stages, studies demonstrate an impact on functionality even in the early stages of CKD (Sridharan et al. 2022; Wilkinson et al. 2025), which shows a gap in research in the early stages. Furthermore, it is noteworthy that responsiveness was not evaluated in any study. Responsiveness is a clinically useful property for evaluating the effect of a therapeutic treatment, which was not the objective or context of any of the studies evaluated. This finding demonstrates another gap in research in individuals with CKD, which is the assessment of the level of PA after therapies.

The present study is also distinguished by using two methodological evaluation tools. The COSMIN initiative is widely used, and it is considered the gold standard in studies related to psychometric properties (Clarke et al. 2018). EMPRO, on the other hand, is a new and still less used tool, but with differentials in its evaluation that make it an interesting option (Prinsen et al. 2018). To date, this is the first published study to use the EMPRO tool for PROMs assessing PA.

COSMIN RoB and EMPRO showed disagreements with each other in the psychometric evaluation of 5 PROMs (45.5%). Rosenkoetter and Tate (2017) observed that the COSMIN RoB items mostly evaluate aspects of the methodological design of psychometric properties, while the EMPRO has a mixed composition of items, including attributes of clinical interest such as application burden and feasibility. However, COSMIN RoB is more robust in guiding usage and discriminating the attributes of each measurement property. Thus, the choice of assessment tool should be based on the researcher's objectives and on their expertise with psychometric assessment.

The present study has some limitations. The research did not include grey literature, but a secondary search was carried out in references of all included studies, as suggested by the COSMIN initiative, to minimize the risk of not including studies not found in databases. The evidence found did not allow discrimination of results by stage of the disease, and the performance of PROMs may differ between stages of CKD. It is important to mention that this study focused on adult individuals and the findings cannot extrapolate to pediatric population.

This systematic review found no consensus in the literature for the best PROM for assessing PA in CKD. CKD‐PAQ appears to be a promising tool as it is the only PROM, with a favorable evaluation by both COSMIN RoB and EMPRO. However, this PROM needs more studies evaluating reliability. This review also found a gap in the evaluation and validation of PROMs in mild stages, as well as the assessment of responsiveness. COSMIN RoB and EMPRO tools differed from each other, showing that the choice of the evaluation tool must be guided by the evaluator's expertise and objective.

### 4.1 Implications for Physiotherapy Practice

Physiotherapists can reduce the impact of functional decline in CKD by assessing and promoting PA in this population. Choosing the best PA assessment tool makes it easier for healthcare professionals to establish functional goals, plan conduct and treatments. PA PROMs are a viable and low‐cost option for clinical use in CKD rehabilitation and monitoring. CKD‐PAQ is a promising tool but lacks reliability and responsiveness studies.

## Author Contributions

T.C.S and L.M.M.S conceptualized the study, developed the research protocol, identified articles, extracted data from studies, synthesized, and analyzed the data. G.A.S analyzed the conflicts among the two reviewers. C.A.G., L.M.M.S and T.C.S discussed the results and contributed to the final manuscript. T.C.S wrote the manuscript. L.M.M.S and I.S.S helped on the manuscript final version. All authors reviewed the manuscript and agreed to its published version.

## Ethics Statement

The authors have nothing to report.

## Consent

The authors have nothing to report.

## Conflicts of Interest

The authors declare no conflicts of interest.

## Permission to Reproduce Material From Other Sources

The authors have nothing to report.

## Supporting information

Supporting Information S1

## Data Availability

The data that support the findings of this study are available from the corresponding author upon reasonable request.
