# Supplementary material for: Properties of Measurements of Patient‐Reported Outcome Measures Physical Activity Assessment in Chronic Kidney Disease: A Systematic Review
Source: Physiother Res Int. 2025 Jun 10;30(3):e70076. doi: 10.1002/pri.70076 (PMC12151139; doi:10.1002/pri.70076)
Supplement: Supplementary file 1 — Supporting Information S1 [file PRI-30-e70076-s001.docx]

**SUPPLEMENTARY INFORMATION**

**Table S1: PRISMA 2020 Checklist**

| **Section and Topic** | **Item #** | **Checklist item** | **Location where item is reported** |
| --- | --- | --- | --- |
| **TITLE** | | |  |
| Title | 1 | Identify the report as a systematic review. | Title page (Article Title) |
| **ABSTRACT** | | |  |
| Abstract | 2 | See the PRISMA 2020 for Abstracts checklist. | Article abstract |
| **INTRODUCTION** | | |  |
| Rationale | 3 | Describe the rationale for the review in the context of existing knowledge. | Article introduction |
| Objectives | 4 | Provide an explicit statement of the objective(s) or question(s) the review addresses. | Article introduction (third paragraph) |
| **METHODS** | | |  |
| Eligibility criteria | 5 | Specify the inclusion and exclusion criteria for the review and how studies were grouped for the syntheses. | Methods (fourth paragraph) |
| Information sources | 6 | Specify all databases, registers, websites, organisations, reference lists and other sources searched or consulted to identify studies. Specify the date when each source was last searched or consulted. | Methods (first paragraph) |
| Search strategy | 7 | Present the full search strategies for all databases, registers and websites, including any filters and limits used. | Methods (second paragraph) and Supplementary File 2 |
| Selection process | 8 | Specify the methods used to decide whether a study met the inclusion criteria of the review, including how many reviewers screened each record and each report retrieved, whether they worked independently, and if applicable, details of automation tools used in the process. | Methods (first and third paragraph) |
| Data collection process | 9 | Specify the methods used to collect data from reports, including how many reviewers collected data from each report, whether they worked independently, any processes for obtaining or confirming data from study investigators, and if applicable, details of automation tools used in the process. | Methods (first and third paragraph) |
| Data items | 10a | List and define all outcomes for which data were sought. Specify whether all results that were compatible with each outcome domain in each study were sought (e.g. for all measures, time points, analyses), and if not, the methods used to decide which results to collect. | Methods (Assessment of the quality of studies section) and Results (Table 1) |
|  | 10b | List and define all other variables for which data were sought (e.g. participant and intervention characteristics, funding sources). Describe any assumptions made about any missing or unclear information. | Not applicable |
| Study risk of bias assessment | 11 | Specify the methods used to assess risk of bias in the included studies, including details of the tool(s) used, how many reviewers assessed each study and whether they worked independently, and if applicable, details of automation tools used in the process. | Methods (Assessment of the quality of studies section) |
| Effect measures | 12 | Specify for each outcome the effect measure(s) (e.g. risk ratio, mean difference) used in the synthesis or presentation of results. | Not applicable |
| Synthesis methods | 13a | Describe the processes used to decide which studies were eligible for each synthesis (e.g. tabulating the study intervention characteristics and comparing against the planned groups for each synthesis (item #5)). | Results (Table 1 and Table 2) |
|  | 13b | Describe any methods required to prepare the data for presentation or synthesis, such as handling of missing summary statistics, or data conversions. | Not applicable |
|  | 13c | Describe any methods used to tabulate or visually display results of individual studies and syntheses. | Results (table 2) |
|  | 13d | Describe any methods used to synthesize results and provide a rationale for the choice(s). If meta-analysis was performed, describe the model(s), method(s) to identify the presence and extent of statistical heterogeneity, and software package(s) used. | Results (table 2) |
|  | 13e | Describe any methods used to explore possible causes of heterogeneity among study results (e.g. subgroup analysis, meta-regression). | Not applicable |
|  | 13f | Describe any sensitivity analyses conducted to assess robustness of the synthesized results. | Not applicable |
| Reporting bias assessment | 14 | Describe any methods used to assess risk of bias due to missing results in a synthesis (arising from reporting biases). | Methods (Assessment of the quality of studies section) |
| Certainty assessment | 15 | Describe any methods used to assess certainty (or confidence) in the body of evidence for an outcome. | Not applicable |
| **RESULTS** | | |  |
| Study selection | 16a | Describe the results of the search and selection process, from the number of records identified in the search to the number of studies included in the review, ideally using a flow diagram. | Results (Figure 1) |
|  | 16b | Cite studies that might appear to meet the inclusion criteria, but which were excluded, and explain why they were excluded. | Results (Figure 1) |
| Study characteristics | 17 | Cite each included study and present its characteristics. | Results (Table 1) |
| Risk of bias in studies | 18 | Present assessments of risk of bias for each included study. | Supplementary information (Files 3 and 4) |
| Results of individual studies | 19 | For all outcomes, present, for each study: (a) summary statistics for each group (where appropriate) and (b) an effect estimate and its precision (e.g. confidence/credible interval), ideally using structured tables or plots. | Results (Table 2) |
| Results of syntheses | 20a | For each synthesis, briefly summarise the characteristics and risk of bias among contributing studies. | Results (Table 2) |
|  | 20b | Present results of all statistical syntheses conducted. If meta-analysis was done, present for each the summary estimate and its precision (e.g. confidence/credible interval) and measures of statistical heterogeneity. If comparing groups, describe the direction of the effect. | Results (Tables 3 and 4) |
|  | 20c | Present results of all investigations of possible causes of heterogeneity among study results. | Not applicable |
|  | 20d | Present results of all sensitivity analyses conducted to assess the robustness of the synthesized results. | Not applicable |
| Reporting biases | 21 | Present assessments of risk of bias due to missing results (arising from reporting biases) for each synthesis assessed. | Not applicable |
| Certainty of evidence | 22 | Present assessments of certainty (or confidence) in the body of evidence for each outcome assessed. | Not applicable |
| **DISCUSSION** | | |  |
| Discussion | 23a | Provide a general interpretation of the results in the context of other evidence. | Discussion (first and second paragraph) |
|  | 23b | Discuss any limitations of the evidence included in the review. | Discussion (second, sixth, seventh and eleventh paragraph) |
|  | 23c | Discuss any limitations of the review processes used. | Discussion (twelfth paragraph) |
|  | 23d | Discuss implications of the results for practice, policy, and future research. | Discussion (eleventh paragraph) |
| **OTHER INFORMATION** | | |  |
| Registration and protocol | 24a | Provide registration information for the review, including register name and registration number, or state that the review was not registered. | Methods (first paragraph) |
|  | 24b | Indicate where the review protocol can be accessed, or state that a protocol was not prepared. | Methods (first paragraph) |
|  | 24c | Describe and explain any amendments to information provided at registration or in the protocol. | Not applicable |
| Support | 25 | Describe sources of financial or non-financial support for the review, and the role of the funders or sponsors in the review. | Title page |
| Competing interests | 26 | Declare any competing interests of review authors. | Title page |
| Availability of data, code and other materials | 27 | Report which of the following are publicly available and where they can be found: template data collection forms; data extracted from included studies; data used for all analyses; analytic code; any other materials used in the review. | Title page |

*From:*  Page MJ, McKenzie JE, Bossuyt PM, Boutron I, Hoffmann TC, Mulrow CD, et al. The PRISMA 2020 statement: an updated guideline for reporting systematic reviews. BMJ 2021;372:n71. doi: 10.1136/bmj.n71

**Table S2. - Descriptors and Boolean operators used in the search strategy**

| **Descriptors disease related** |  | **Descriptors construct related** |  | **Descriptors psychometric related** |
| --- | --- | --- | --- | --- |
| Chronic Kidney Disease  **OR**  Chronic Renal Insufficiency  **OR**  Chronic Renal Disease  **OR**  Dialysis | **AND** | Physical Activity  **OR**  Physical Activities | **AND** | Psychometrics  **OR**  Patient Outcome Assessment  **OR**  Outcome Assessment, Health Care  **OR**  Reproducibility of Results  **OR**  Reliability of Results  **OR**  Validity of Results  **OR**  Reliability and Validity  **OR**  Test Retest Reliability  **OR**  Validity, Face  **OR**  Finding Reproducibility  **OR**  Validation studies  **OR**  Cross Cultural Comparison |

**Table S3. Search strategy for each database**

| **PubMed / Medline** | (chronic renal insufficiency [MeSH Terms] OR "chronic kidney disease" OR "chronic renal disease" OR "dialysis") AND (physical activity [MeSH Terms] OR "physical activities") AND (psychometrics [MeSH Terms] OR "patient outcome assessment" OR "outcome assessment, health care" OR "reproducibility of results" OR "reliability of results" OR "validity of results" OR "reliability and validity" OR "test retest reliability" OR "validity, face" OR "finding reproducibility" OR "validation studies" OR "cross cultural comparison") |
| --- | --- |
| **Lilacs** | (mh:"chronic renal insufficiency" OR tw:"chronic kidney disease" OR tw:"chronic renal disease" OR tw:"dialysis") AND (mh:"physical activity" OR tw:"physical activities") AND (mh:"psychometrics" OR "patient outcome assessment" OR "outcome assessment, health care" OR tw:"reproducibility of results" OR tw:"reliability of results" OR tw:"validity of results" OR tw:"reliability and validity" OR tw:"test retest reliability" OR tw:"validity, face" OR tw:"finding reproducibility" OR tw:"validation studies" OR tw:"cross cultural comparison") |
| **SciELO** | (mh:"chronic renal insufficiency" OR tw:"chronic kidney disease" OR tw:"chronic renal disease" OR tw:"dialysis") AND (mh:"physical activity" OR tw:"physical activities") AND (mh:"psychometrics" OR "patient outcome assessment" OR "outcome assessment, health care" OR tw:"reproducibility of results" OR tw:"reliability of results" OR tw:"validity of results" OR tw:"reliability and validity" OR tw:"test retest reliability" OR tw:"validity, face" OR tw:"finding reproducibility" OR tw:"validation studies" OR tw:"cross cultural comparison") |
| **EMBASE** | ('chronic renal insufficiency'/exp OR 'chronic renal insufficiency' OR 'chronic kidney disease'/exp OR 'chronic kidney disease' OR 'chronic renal disease'/exp OR 'chronic renal disease' OR 'dialysis'/exp OR 'dialysis') AND ('physical activity'/exp OR 'physical activity' OR 'physical activities'/exp OR 'physical activities') AND ('psychometrics'/exp OR 'psychometrics' OR 'patient outcome assessment' OR 'outcome assessment, health care' OR 'reproducibility of results':ti,ab OR 'reliability of results':ti,ab OR 'validity of results':ti,ab OR 'reliability and validity':ti,ab OR 'test retest reliability':ti,ab OR 'validity, face':ti,ab OR 'finding reproducibility':ti,ab OR 'validation studies':ti,ab OR 'cross cultural comparison':ti,ab) |

| **Table S4. Analysis of the methodological quality of studies using COSMIN RoB.** | | | | | | | | | | |
| --- | --- | --- | --- | --- | --- | --- | --- | --- | --- | --- |
| **Study** | **PROM** | **Psychometric properties** | | | | | | | | |
|  |  | **Construct validity (structural)** | **Construct validity (hypotheses testing)** | **Content validity** | **Criterion validity** | **Convergent validity** | **Cross-cultural validity** | **Internal consistency** | **Reliability** | **Measurement error** |
| Hadjiioannou et al., 2019 | DASI | - | - | - | - | - | - | - | Adequate | Adequate |
| Hatef et al., 2018 | ESES | Very good | - | Doubtful | - | Very good | Adequate^a^ | Very good | Doubtful | - |
| Huang et al., 2021 | LoPAQ | - | - | Doubtful | Very good | - | Inadequate^b^ | Inadequate | Adequate | - |
| Johansen et al., 2001 | HAP  PAR  PASE | - | - | - | Very good | - | - | - | Inadequate | - |
| Johansen et al., 2015 | LoPAQ | - | Very good | - | - | Very good | - | - | - | - |
| Kittiskulnam et al., 2019 | LoPAQ | - | Adequate | - | Very good | - | - | - | - | - |
| Lou & He, 2019 | IPAQ | - | - | - | Very good | - | - | - | Adequate | - |
| Overend et al., 2010 | HAP | - | - | - | - | - | - | - | Adequate | Adequate |
| Ravani et al., 2012 | DASI | - | - | - | Very good | - | - | - | Adequate | - |
| Robinson-Cohen et al., 2013 | FWH  HAP  IPAQ^†^  PASE | - | - | - | Very good | - | - | - | - | - |
| Rosa et al., 2015 | IPAQ | - | - | - | Very good | - | - | - | - | - |
| Sridharan et al., 2016 | PAR  RPAQ | - | - | - | Very good | - | - | - | - | - |
| Sridharan et al., 2022 | CKD-PAQ | - | Adequate | - | - | Adequate | - | - | - | - |
| Tabib et al., 2024 | LoPAQ | - | - | Doubtful | - | Very good | Doubtful^a^ | - | Adequate | - |
| Wellard, 2003 | HAP^†^ | - | - | - | - | Adequate | - | - | - | - |
| Wilkinson et al., 2020 | GPPAQ | - | - | - | Very good | - | - | - | Adequate | - |
| Yamabe et al., 2023 | LoPAQ | - | - | - | Very good | - | Adequate^c^ | - |  | - |
| ^†^tool-specific domain analysis; ^a^Regarding the cross-cultural validation of PROM in Persian; ^b^Regarding the cross-cultural validation of PROM in Mandarin; ^c^Regarding the cross-cultural validation of PROM in Japanese. | | | | | | | | | | |

| **Table S5. Methodological evaluation of the studies included by the EMPRO tool.** | | | | | | | | | | |
| --- | --- | --- | --- | --- | --- | --- | --- | --- | --- | --- |
| **Study** | **PROM** | **EMPRO attributes** | | | | | | | | |
|  |  | **Conceptual and measurement model** | **Reliability** | | **Validity** | **Responsiveness** | **Interpretabilty** | **Burden** | **Alternative modes of administration** | **Cultural and language adaptations or translations** |
|  |  |  | Internal consistency | Reproducibility |  |  |  |  |  |  |
| Hadjiioannou et al., 2019 | DASI | - | 33.3 | 83.3* | - | - | - | - | - | - |
| Hatef et al., 2018 | ESES | 71.4* | 58.3* | 16.7 | 38.9 | - | - | 22.2 | - | 100.0*(a) |
| Huang et al., 2021 | LoPAQ | - | 8.3 | 25.0 | 33.3 | - | - | - | - | 88.9*(b) |
| Johansen et al., 2001 | HAP  PAR  PASE | - | - | 33.3 | 5.5 | - | 55.5* | - | - | - |
| Johansen et al., 2015 | LoPAQ | 66.7* | - | - | 16.6 | - | 55.5* | - | - | - |
| Kittiskulnam et al., 2019 | LoPAQ | - | - | - | 33.3 | - | 66.6* | - | - | - |
| Lou & He, 2019 | IPAQ | - | - | 50.0* | 5.5 | - | - | - | - | - |
| Overend et al., 2010 | HAP | - | 16.6 | 66.6* | - | - | 55.5* | - | - | - |
| Ravani et al., 2012 | DASI | - | - | 50.0* | 22.2 | - | 55.5* | - | - | - |
| Robinson-Cohen et al., 2013 | FWH  HAP  IPAQ^†^  PASE | - | - | - | 22.1 | - | 44.4 | - | - | - |
| Rosa et al., 2015 | IPAQ | - | - | - | 11.1 | - | - | - | - | - |
| Sridharan et al., 2016 | PAR  RPAQ | - | - | - | 22.2 | - | 77.7* | - | - | - |
| Sridharan et al., 2022 | CKD-PAQ | 52.4* | - | - | 50.0* | - | 55.5* | - | - | - |
| Tabib et al., 2024 | LoPAQ | - | - | 33.3 | 44.4 | - | - | - | - | 66.6*(a) |
| Wellard, 2003 | HAP^†^ | - | - | - | 11.1 | - | - | - | - | - |
| Wilkinson et al., 2020 | GPPAQ | - | - | 58.3* | 50.0* | - | 66.6* | - | - | - |
| Yamabe et al., 2023 | LoPAQ | - | - | - | 72.2* | - | - | - | - | 44.4 (c) |
| *Acceptable value; ^†^tool-specific domain analysis; (a)Regarding the cross-cultural validation of PROM in Persian; (b)Regarding the cross-cultural validation of PROM in Mandarin; (c)Regarding the cross-cultural validation of PROM in Japanese. | | | | | | | | | | |
